# Paths to cheminformatics: Q&A with Ann M. Richard

**DOI:** 10.1186/s13321-023-00749-1

**Published:** 2023-10-05

**Authors:** Ann M. Richard

**Affiliations:** The U.S. Environmental Protection Agency, Durham, NC USA

## Introduction by the Editors-in-Chief

Recently we described [1] an initiative to put a spotlight on diversity within the cheminformatics community. As part of that we initiated a series of interviews, and this article continues that series. Prior to her retirement in March of this year, Ann M. Richard, PhD, worked as a Research Chemist at the U.S. Environmental Protection Agency (EPA) for the entirety of her 36-year professional career. Her research during that time spanned applications of quantum chemistry to modeling mechanisms of toxicity, building a public chemical structure–toxicity database (DSSTox) to support structure–activity relationship (SAR) modelers, leading the chemical management of EPA’s transformative ToxCast and Tox21 high-throughput screening (HTS) programs, helping to design EPA’s CompTox Chemicals Dashboard, and developing novel chemotype-enrichment approaches to model inherently noisy, HTS data generated for thousands of environmentally relevant chemicals. She will be returning as an Emeritus scientist at EPA to continue to serve as a resource, mentor, and scientific collaborator for the foreseeable future.

## What has been your path to where you are today?

AMR: First in my family to graduate from college (SUNY-Oswego) in 1978, I dual majored in Chemistry and Math. My future husband and I moved to North Carolina to each attend graduate school in Chemistry at UNC-Chapel Hill, hardly believing we would be paid to do this. My graduate work was in Theoretical Physical Chemistry, bridging quantum and classical approaches to model small molecule energy transfer in the gas phase and at solid surface interfaces. Mainframe computers were just becoming accessible with remote terminals, personal computers were in their infancy, and the Internet was several years off by the time I graduated in 1983; additionally, there were very few women in my field. Shortly after, I was hired as a post doc in EPA’s National Health & Environmental Effects Research Lab working under Dr. James Rabinowitz. My research focused on extending a technique for efficiently computing electrostatic potentials to model chemical similarity of small molecules. I joined EPA as a Principal Investigator in 1987 and over the next decade established collaborations with toxicologists and experimentalists across EPA, applying computational chemistry approaches to elucidate mechanisms and build SAR models [2, 3]. During that time, I came to appreciate the challenges and rewards of applying these approaches to real-world problems in toxicology. I gained recognition as an effective (and passionate) communicator to cross-disciplinary audiences, relating important details while mindful of the bigger picture, which opened doors and opportunities to me as a young scientist. Being a peacemaker by nature, I was also stepping into the role of neutral arbiter in evaluating and critiquing the new expert-based and computational global SAR models (mostly commercial software) being applied to predicting mutagenicity and carcinogenicity [4–6]. I came to understand the critical role of quality training sets for building such models, the challenges of modeling mechanistical complex toxicity endpoints (vs. physicochemical properties or a receptor target activity), and how the commercial “black box” software applications were built on business models that mined public data and then sequestered the resulting training sets as proprietary. I began to strongly advocate for a public data sharing approach to ensure model transparency, quality, and reproducibility, as well as to help the field progress. At the same time, I became increasingly aware of the many siloed, independently maintained, chemical lists lacking structures across EPA’s Program Offices that were supporting regulatory programs such as the Clean Water Act, Clean Air Act, Superfund, etc. In the last slide of an invited presentation at the 2000 QSAR meeting in Burgas, Bulgaria, I proposed development of a public, web-based, standardized, and curated structure–toxicity data resource to serve the SAR toxicity modeling community; there were no publicly available structure databases at that time. With the help of a talented student contractor (C. Williams-Devane), I adopted the motto “Just do it” and published the initial DSSTox database and public website in 2004, helping to pierce some of EPA’s early barriers to open science [7, 8]. I was recruited to EPA’s newly formed National Center for Computational Toxicology (NCCT) in 2005 and received a small grant to hire a full time DSSTox chemical curator (M. Wolf) and develop EPA’s first and only (until 2021) web-based, structure-similarity search capability in association with the DSSTox website. As one of the only chemists in NCCT, I took on contract manager responsibilities for procuring, solubilizing, plating, and data management of thousands of chemicals to support EPA’s new ToxCast and Tox21 programs, which were adopting HTS technologies of the pharmaceutical industry to transforming toxicology. I accepted this multi-year, support-role, convinced that DSSTox curation and strict chemical quality control measures would be necessary to ensure the success of toxicity prediction models using HTS data. I further helped to design and develop the cheminformatics infrastructure to support these programs, while advocating for an integration of SAR concepts and HTS data to advance predictive toxicology [9, 10]. Dr. Chihae Yang, a long-time collaborator and supporter, was a leader in the area of toxicity data informatics and modeling during this time, not only advocating for, but doing the hard work herself of curating toxicity datasets for use in modeling [11, 12]. She and her colleagues at Molecular Networks GmbH, with a grant from the U.S. Food & Drug Administration, publicly released an expert-derived, knowledge-informed set of public ToxPrint fingerprints for use in toxicity modeling in 2013 [13], which helped to launch the next chapter of my research. Employing ToxPrints, I developed a simple, standardized chemotype-enrichment approach to help ToxCast researchers better understand and elucidate chemical structure patterns within and across their HTS datasets and, in the process, came to better appreciate the complexity and challenges associated with these datasets [14, 15]. Around the same time, partnering with the extraordinary talented duo of Drs. Chris Grulke and Antony Williams in 2014 and 2015, the original, manually curated DSSTox database was migrated to MySQL and greatly expanded, becoming the underpinning for EPA’s CompTox Chemicals Dashboard (https://comptox.epa.gov/dashboard/), launched in 2017 [16, 17]. The latter currently hosts over 1.2 M DSSTox substances (> 1 M structures) linked to hundreds of regulatory and data lists, spanning HTS, toxicity, hazard, and exposure, and is supporting EPA and environmental researchers worldwide.

## What is your current research focus, and what are your plans for the future?

AMR: A few years prior to retiring earlier this year, I had shifted my research efforts to focus on PFAS (per- and poly-fluoroalkyl substances), an emerging contaminant problem area for EPA. This entailed supervising the DSSTox curation of thousands of PFAS substances (represented by both defined structures and Markush) from public sources [18], procurement and plating of an actual PFAS test library of more than 400 substances being screened in phases in a subset of ToxCast assays, and, most recently, development of a customized set of PFAS fingerprints (an extension of the public ToxPrints) to support cheminformatics research and modeling in this chemical domain [19]. I also helped to integrate the ToxPrint chemotype-enrichment approach, as well as the results from global analysis of more than 1000 ToxCast assays, into a public, on-line tool (Cheminformatics Modules: https://www.epa.gov/chemical-research/cheminformatics). Through seminars and individual collaborations, I trained several of EPA’s younger scientists in use of the approach, as well as use of the customized PFAS fingerprints. As an Emeritus, I plan to continue to support the thoughtful application of computational and cheminformatics approaches, advocate for quality DSSTox structure curation to complement testing and modeling efforts, and provide guidance and encouragement to EPA’s next generation of researchers.

## Which obstacles did you encounter during your career, and what experiences have helped you get to where you are today?

AMR: Starting my career at EPA in a multidisciplinary team of toxicologists, molecular biologists, and life scientists, my challenge early on was a steep learning curve and finding where I could best apply my training and skills to support fellow researchers and EPA’s mission. The biggest obstacle was gaining the trust and respect of my experimental toxicology colleagues, and advocating for myself. This was a slow, iterative process, where I took the time to listen, learn, and understand enough of their experiments and data to gauge where and how a computational chemistry approach could contribute to further understanding [1]. Early on, the onus was most often on me to cross the learning bridge, articulate a hypothesis, and initiate the collaboration. However, each time a project met with success, I gained an enthusiastic collaborator and advocate who was eager to embrace these approaches in future collaborations. This success even extended to early interactions with a new journal editor (L. Marnett, Chemical Research in Toxicology), who was initially skeptical of structure-based toxicity prediction models, as they were often divorced from mechanistic interpretation. However, he came to appreciate the potential and value of models and computational experiments, when thoughtfully constructed, to elucidate mechanisms in toxicology [3]. I also put a lot of thought and effort into communicating computational approaches and results, in writing and in presentations, in a way that was respectful of diverse audiences, entertaining (colorful graphics, minimizing text), and easy to digest and grasp. To this day, the many hours I have devoted to creating information dense, colorful graphics for conveying complex ideas and results in clear, simple terms has served me well [10, 20]. Lastly, I want to say that pursuing a career in a government research laboratory, supporting a public health mission, has been very enabling and rewarding—I do not believe that I could have charted my research path or had the impact that I have had in my career elsewhere in industry or academia. In addition, my government job provided me the opportunity to be part of a community of extraordinarily committed and talented scientists, travel, teach, and pursue my scientific interests, all while enabling a work-life balance that included raising a family and twice being a caregiver to elderly parents. As a woman in the physical sciences, I feel that we have come a long way since 1983, but that balancing work and family caregiver responsibilities (for both young and old) is still challenging for women in science.

## What advice would you give to your younger self?

AMR: Firstly, do not apologize for being emotional when talking about things that you care deeply about—it is not something you can control and is just who you are; if it makes managers uncomfortable, that is their problem; if they think less of you for it, push through and prove them wrong. Secondly, continue to strongly advocate for what you believe in and care about—passion and truth are your fuel and will help to overcome many obstacles and inspire others. The term cheminformatics will be coined and eventually recognized as a valid and transformative scientific discipline, and you will be joined in your data curation/informatics efforts and supported by a community of scientists who understand and share your vision and commitment. Stay the course in your efforts to change entrenched attitudes about the importance of quality chemistry databases to support modeling and, just remember—the slow, deliberate turtle with the hard shell ultimately wins the race against the fast and careless rabbit.

## What is a current challenge you are facing that should not be a challenge in the near future?

AMR: As more than one prior interviewee has said or implied, data is the biggest enabler and limiting factor for cheminformatics modelers. My career trajectory took a major turn once I recognized the pivotal role that quality chemical representations and their accurate association with activity data had to play in achieving predictive toxicology objectives. Although collecting, cleaning, and curating data and list associations is a tedious and time-consuming pursuit (and looked down on by some as not sufficiently scientific or innovative), it is something that almost every modeler has had to tackle at some point. In the process, one comes to understand the value and limitations of those data, and realize that quality data, in turn, can improve the science of the modeling enterprise. As I shifted my focus to cleaning up datasets and chemical structure-identifier associations, I became alarmed at the scope of the problem across public datasets, undermining toxicity modeling efforts. Expert manual chemistry curation in association with bioactivity data, which only needs to be done right once and then publicly shared, is the most effective solution, which is why I took on this challenge. I am also convinced that if the toxicology research community does not get the chemistry right, we modelers are hobbled right out of the starting gate. Consider, e.g., a multi-million dollar, 2-year rodent carcinogenicity study published with a chemical name and CAS RN that point to different substances, or where the structure is wrong or important stereochemistry is missing or ambiguous. Or consider a chemical-activity HTS result where the chemical was originally misidentified by a major supplier (yes, this happens) or, unbeknownst to the experimenter, the chemical reacted, volatilized, or degraded under the testing conditions. Additionally, I have learned first-hand the extent to which testing artifacts (both chemical and biological) can obscure the desired target activity, resulting in misleading modeling outcomes. Too often, modelers scour the Internet for chemical-activity data sets, and apply the newest modeling approach without doing the work to clean and understand the data or endpoint being modeled. I also well understand the economic rationale behind the pharmaceutical and chemical industries’ reluctance to publicly share toxicity data and knowledge that is intertwined with the pursuit of new drugs. However, failure to predict drug toxicity is a major impediment to new drug advances, just as it is a challenge from a public health standpoint. My hope for the near future is that public data resources supporting chemical-toxicity evaluation and regulation not only continue to expand, but that quality chemical curation becomes the norm and is demanded and expected by the scientific and regulatory communities.

## What do you think the cheminformatics community could do to increase diversity and inclusion?

AMR: I would like to see increased opportunities for younger scientists to more actively participate and contribute to public scientific forums, not just giving presentations but in facilitated discussions. I continue to see too many younger, mostly women scientists (particularly minorities and Asians) seated on the periphery of conference rooms, seemingly afraid to speak up or ask questions, particularly when a small number of senior scientists (not always, but most often male) dominate the discussion. Until the scales are truly balanced to include diverse voices, these younger scientists need to be actively encouraged and nudged by those same senior scientists to sit at that table, ask questions, and contribute to the discussion. With the growing availability of “big data” and increasingly sophisticated machine-learning and AI methods, cheminformatics is a rapidly evolving discipline. However, it remains highly multi-disciplinary and requires engagement with, and understanding of the data being modeled. Younger scientists have a lot to learn, but they also bring a fresh outlook and new skills to contribute to the field. They should be actively encouraged to engage in continuous learning to broaden their perspective, sit at the table, and speak up. It is the responsibility of the older generation to guide, encourage, and make this path easier.

## What is your thought on ChatGPT/Large Language Models and how these might influence the way we do science?

AMR: I have started seeing colleagues investigate ChatGPT to compile short biographies and abstracts covering various scientific topics and fields of studies. The results have been impressive, sometimes humorous, and mostly (but not always) accurate, largely due to the volume of publicly available scientific information in the form of open-access articles, PubMed abstracts, and proceedings of scientific meetings. I can also see large language models being potentially useful for improving context recognition in text data mining, if fed or pointed to appropriate and trustworthy resources. Additionally, they might be helpful for non-English speakers in writing and editing scientific manuscripts for submission to English journals. Some issues that need to be grappled with, however, are how journals and others will use, oversee, and require disclosure of AI-generated text. Without adequate oversight, it has already been shown that ChatGPT can create a convincing looking scientific abstract or article, right down to the fake references (https://www.biorxiv.org/content/10.1101/2022.12.23.521610v1). This presents the unscrupulous with a ripe opportunity to spread misinformation with a scientific veneer. Within the cheminformatics community, chemistry is already our lingua franca and ChatGPT is just a next iteration of AI, which has been used in various forms (machine-learning algorithms, neural nets, etc.) in this area for many years now. Focusing on the positives, AI can be a powerful tool for advancing the science if harnessed not only to quality data, but to the iterative scientific enterprise to ensure that results inform, illuminate, and guide a path forward. These are powerful techniques that can serve our field but they need to be tethered to critical review, understanding, and analysis. A chilling recent experiment with these methods points to unanticipated, possible negative applications: Ekins and coworkers [21] reported reversing the drug discovery AI paradigm to discover potentially potent chemical weapons (the work is also featured in a new Netflix documentary titled “Unknown: Killer Robots”). As with researchers in many other fields grappling with the impact of these new approaches, the cheminformatics community needs to keep their eyes wide open to both the potential and pitfalls of the approaches.

## Photos

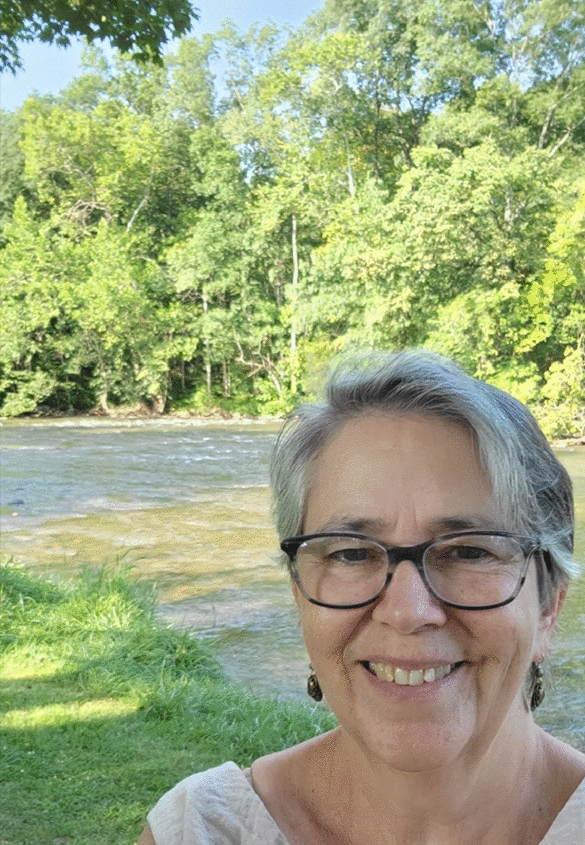



## Data Availability

Not applicable.

## References

[1] Zdrazil B, Guha R (2022). Diversifying cheminformatics. J Cheminform.

[2] Richard AM, Hunter ES (1996). Quantitative structure-activity relationships for the developmental toxicity of haloacetic acids in mammalian whole embryo-culture. Teratology.

[3] Shim JY, Richard AM (1997). Theoretical evaluation of two plausible routes for bioactivation of S-(1,1-difluoro-2,2-dihaloethyl)-L-cysteine conjugates: thiirane vs. thionoacyl fluoride pathway. Chem Res Toxicol.

[4] Richard AM (1994). Application of SAR methods to non-congeneric data bases associated with carcinogenicity and mutagenicity: issues and approaches. Mutat Res.

[5] Richard AM (1998). Structure-based methods for predicting mutagenicity and carcinogenicity: are we there yet?. Mutat Res.

[6] Richard AM (1999). Application of artificial intelligence and computational methods to predicting toxicity. Knowledge Eng Rev.

[7] Richard AM, Williams CR (2002). Distributed structure-searchable toxicity (DSSTox) database network: a proposal. Mutat Res.

[8] Richard AM, Gold LS, Nicklaus MC (2006). Chemical structure indexing of toxicity data on the internet: moving towards a flat world. Curr Opin Drug Discov.

[9] Richard AM (2006). Future of predictive toxicology: an expanded view of “chemical toxicity”—future of toxicology perspective. Chem Res Toxicol.

[10] Richard AM, Judson RS, Houck KA, Grulke CM, Volarath P, Thillainadarajah I, Yang C, Rathman J, Martin MT, Wambaugh JF, Knudsen TB, Kancherla J, Mansouri K, Patlewicz G, Williams AJ, Little SB, Crofton KM, Thomas RS (2016). The ToxCast chemical landscape: paving the road to 21st century toxicology. Chem Res Toxicol.

[11] Richard A, Yang C, Judson R (2008). Toxicity data informatics: supporting a new paradigm for toxicity prediction. Tox Mech Meth.

[12] Yang C, Cronin MTD, Arvidson KB, Bienfait B, Enoch S, Heldreth B, Hobocienski B, Lan Y, Madden J, Magdirarz T, Marusczyk J, Mostrag A, Nelms M, Neagu D, Przybylak K, Rathman J, Park J, Richarz A-N, Richard AM, Ribiero V, Sacher O, Schwab C, Vitcheva V, Volarath P, Worth AP (2021). COSMOS DB & next generation (NG): a database and knowledge hub foundation to leverage public resources in collaboration with regulatory offices for cosmetics, food ingredients, and biological data. Computat Toxicol.

[13] Yang C, Tarkhov A, Marusczyk J, Bienfait B, Gasteiger J, Kleinoeder T, Magdziarz T, Sacher O, Schwab CH, Schwoebel J, Terfloth L, Arvidson K, Richard A, Worth A, Rathman J (2015). New publicly available chemical query language, CSRML, to support chemotype representations for application to data mining and modeling. J Chem Inf Model.

[14] Strickland JD, Martin MT, Richard AM, Houck KA, Shafer TJ (2018). Screening the ToxCast Phase II libraries for alterations in network function using cortical neurons grown on multi-well microelectrode array (mwMEA) plates. Arch Toxicol.

[15] Wang J, Richard AM, Murr AS, Buckalew AR, Lougee RR, Shobair M, Hallinger DR, Laws SC, Stoker T (2021). Expanded high-throughput screening and chemotype-enrichment analysis of the Phase II:e1k ToxCast library for human sodium-iodide symporter (NIS) inhibition. Arch Toxicol.

[16] Williams A, Grulke CM, Edwards J, McEachran A, Mansouri K, Baker NC, Patlewicz G, Shah I, Wambaugh JF, Judson R, Richard AM (2017). The CompTox chemistry dashboard—a community data resource for environmental chemistry. J Chem Inf.

[17] Grulke CM, Williams AJ, Thillainadarajah I, Richard AM (2019). EPA’s DSSTox chemical structure database: a curated chemistry resource supporting computational toxicology research. Comput Toxicol.

[18] Williams AJ, Gaines LG, Grulke C, Lowe CN, Sinclair G, Samano V, Thillainadarajah I, Meyer B, Patlewicz G, Richard AM (2002). Assembly and curation of lists of per-and polyfluoroalkyl substances (PFAS) to support environmental science research. Front Environ Sci.

[19] Richard AM, Lougee R, Adams M, Hidle H, Yang C, Rathman J, Magdziarz T, Williams AJ, Patlewicz G (2023). A new CSRML structure-based fingerprint method for systematically profiling and categorizing Per- and Polyfluoroalkyl substances (PFAS). Chem Res Toxicol.

[20] Richard AM, Huang R, Waidyanatha S, Shinn P, Collins BJ, Thillainadarajah I, Grulke CM, Williams AJ, Lougee RR, Judson RS, Houck KA, Shobair M, Yang C, Rathman JF, Yasgar A, Fitzpatrick SC, Simeonov A, Thomas RS, Crofton KM, Paules RS, Bucher JR, Austin CP, Kavlock RJ, Tice RR (2021). The Tox21 10K compound library: collaborative chemistry advancing toxicology. Chem Res Toxicol.

[21] Urbina F, Lentzos F, Invernizzi C, Ekins S (2022). Dual use of artificial-intelligence-powered drug discovery. Nat Mach Intell.

